# Diagnostic Biomarkers for Pancreatic Ductal Adenocarcinoma Using Non-Targeted Metabolomic Analysis

**DOI:** 10.3390/cancers18040684

**Published:** 2026-02-19

**Authors:** Hirofumi Sonoda, Hideo Ogiso, Yuichi Aoki, Kazue Morishima, Hideki Sasanuma, Naohiro Sata, Joji Kitayama, Hiroharu Yamashita, Hironori Yamaguchi, Ryozo Nagai, Kenichi Aizawa

**Affiliations:** 1 Department of Surgery, Jichi Medical University, Tochigi 329-0498, Japan; 2 Department of Translational Research, Clinical Research Center, Jichi Medical University Hospital, Tochigi 329-0498, Japan; 3 Division of Clinical Pharmacology, Department of Pharmacology, Jichi Medical University, Tochigi 329-0498, Japan; 4 Jichi Medical University, Tochigi 329-0498, Japan; 5 Clinical Pharmacology Center, Jichi Medical University Hospital, Tochigi 329-0498, Japan

**Keywords:** pancreatic ductal adenocarcinoma, pancreatic juice, metabolomics, diagnostic biomarker, liquid biopsy

## Abstract

**Background**: Liquid biopsy using bodily fluids enables noninvasive acquisition of diverse tumor-derived molecules for comprehensive characterization of tumor profiles. Metabolomic analysis, in particular, may accurately reflect disease pathogenesis and holds promise for clinical diagnostic applications. **Objective:** This study explored metabolic alterations associated with pancreatic ductal adenocarcinoma (PDAC) using non-targeted metabolomic analysis of pancreatic juice to construct a preliminary diagnostic model based on selected metabolites. **Methods:** Pancreatic juice samples were collected intraoperatively and postoperatively from patients undergoing pancreaticoduodenectomy for PDAC (*n* = 11) and from those who had non-PDAC diseases, including benign conditions such as chronic pancreatitis and non-pancreatic malignancies such as distal bile duct adenocarcinoma and ampullary adenocarcinoma (*n* = 14). Non-targeted metabolomic analysis was performed using LC-QTOF-MS. Data were processed using MS-DIAL and MetaboAnalyst, and components showing intergroup differences were selected via PLS-DA. A diagnostic model was constructed using logistic regression based on annotated metabolites. **Results:** PLS-DA identified 56 discriminative components, of which 19 were successfully annotated. One metabolite was notably increased and 22 were relatively decreased in pancreatic juice of patients with PDAC. Among known metabolites that tended to decrease were isocitric acid, citric acid, and several oxidized fatty acids. A tentative logistic regression-based diagnostic model using these selected metabolites showed moderate discriminative performance. Citric acid was included in the final three-variable model, suggesting its potential as a candidate marker for PDAC discrimination. **Conclusions:** Pancreatic juice reflects PDAC-associated metabolic changes and may contain candidate diagnostic biomarkers. Metabolites annotated in this study may have potential as novel markers, and further studies on unknown components could help advance PDAC diagnosis and treatment.

## Simple Summary

Bodily fluids of cancer patients contain various tumor-derived molecules that can provide a broad overview of cancer status. This study analyzed pancreatic juice using non-targeted metabolomic profiling to identify characteristic metabolic changes associated with pancreatic ductal adenocarcinoma (pancreatic cancer) and to develop a provisional diagnostic model. We analyzed pancreatic juice from 11 patients with pancreatic cancer and 14 patients with other benign or malignant diseases, including chronic pancreatitis and non-pancreatic malignancies such as distal bile duct adenocarcinoma and ampullary adenocarcinoma, extracting 56 metabolites that differentiated the two groups. Of these, 19 were annotated. One metabolite was notably increased and 22 were relatively decreased in pancreatic cancer. Among the decreased metabolites were isocitric acid, citric acid, and oxidized fatty acids. Using annotated metabolites, we constructed a logistic regression diagnostic model that demonstrated moderate discriminatory ability. Citric acid was included in the final, three-metabolite model, suggesting its potential usefulness as a diagnostic marker. These findings indicate that metabolic signatures in pancreatic juice may enable development of new diagnostic approaches for pancreatic cancer.

## 1. Introduction

Pancreatic ductal adenocarcinoma (PDAC) has extremely poor prognosis, with a five-year survival rate markedly lower than that of other solid tumors [1,2,3,4]. PDAC mortality ranks fourth among men, following lung, prostate, and colorectal cancers, and third among women, following lung and breast cancers [4,5]. Surgical resection remains the most reliable therapeutic option; thus, efficient detection of resectable PDAC and early therapeutic intervention are crucial to reduce disease-related mortality [6,7]. However, early detection is particularly challenging due to the absence of symptoms in initial stages and the lack of established screening programs specific to PDAC [8,9].

Diagnostic tests for PDAC include imaging and endoscopic examinations, but recent technological advances are enabling earlier diagnosis [10]. When PDAC is suspected based on contrast-enhanced Computed Tomography (CT) scan results, Magnetic Resonance Cholangiopancreatography (MRCP), endoscopic ultrasound, and Endoscopic Retrograde Cholangiopancreatography (ERCP) are performed. However, by the time these tests are employed, there is a high risk of advanced PDAC and the window for surgical resection may already have passed. Furthermore, performing these tests as screening for PDAC increases costs and burdens on examiners and patients, due to the time and invasive nature of these tests [11]. Currently, serum biomarkers such as CA19-9 and CEA are used clinically. However, their sensitivity and specificity are insufficient to diagnose PDAC, and false-negative results remain a clinical issue in Lewis antigen-negative patients [12]. In addition, CA19-9 and CEA are not tumor-specific biomarkers and can be elevated in various non-neoplastic conditions, including inflammatory and benign diseases, which further limits their diagnostic utility [13,14]. To address these issues, liquid biopsies, which measure biomarkers in bodily fluids such as blood, are becoming more widely used because they are minimally invasive and rapid.

Liquid biopsy enables detection of tumor-specific molecular signatures, by sampling extracellular vesicles such as exosomes [15], circulating DNA [16], RNA [17,18,19], microRNAs [20], tumor-associated proteins [21,22,23], and lipids [24]. This approach provides a comprehensive molecular profile of tumors, offering potential applications in diagnosis, therapeutic selection, and assessment of treatment response [25,26,27]. Advances in next-generation sequencing technologies have facilitated comprehensive analyses of nucleic acid expression patterns, such as cell-free DNA and microRNA in bodily fluids, and these approaches are increasingly being integrated into clinical practice for cancer diagnostics [28,29,30]. Nevertheless, beyond genomic information, proteomic and metabolomic analyses have substantial diagnostic value. In this study, we focused on non-targeted metabolomics and conducted analyses aimed at identifying candidate metabolic features relevant to the diagnosis of pancreatic cancer.

Non-targeted metabolomics is a technique for comprehensively analyzing metabolites with molecular weights of ~900 Da or less in biological systems, while highlighting those that contribute most significantly to various processes [31]. Based on high-performance liquid chromatography coupled with high-resolution mass spectrometry (LC-MS), this approach has emerged as a powerful tool for exploring metabolic changes in samples under different conditions, with great potential in fields such as disease diagnosis [32,33,34,35]. In this study, we performed non-targeted metabolomic analysis based on LC-MS using stored pancreatic juice samples to reveal differences in metabolites between two patient groups, PDAC and non-PDAC diseases, including benign conditions such as chronic pancreatitis and non-pancreatic malignancies such as distal bile duct adenocarcinoma and ampullary adenocarcinoma. Metabolites differentiating the two groups were selected using partial least-squares discriminant analysis (PLS-DA). Causes of serum metabolic changes associated with PDAC are discussed.

## 2. Materials and Methods

### 2.1. Ethics Statement

The present study was approved by Jichi Medical University Clinical Research Ethics Committee (Approval No. 24-122). Informed consent was obtained from all participants in accordance with the Declaration of Helsinki.

### 2.2. Patients

This study involved patients who underwent pancreaticoduodenectomy for pancreatic cancer and other benign or malignant diseases, including chronic pancreatitis and non-pancreatic malignancies such as distal bile duct adenocarcinoma and ampullary adenocarcinoma, at the Department of Gastrointestinal Surgery, Jichi Medical University, between April 2024 and December 2024. Pancreatic juice samples were collected from patients who provided written informed consent prior to surgery.

### 2.3. Sample Collection

**Figure 1:**
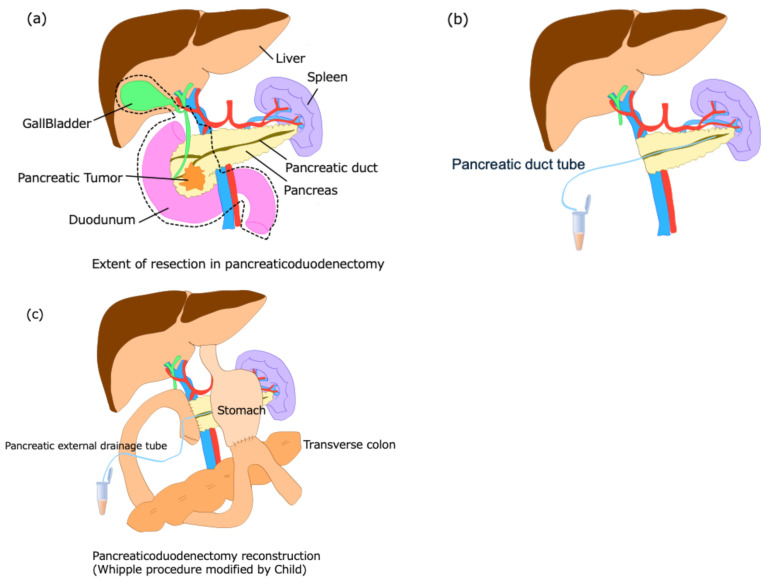
Pancreaticoduodenectomy procedure and collection of pancreatic juice. (**a**) Extent of resection in pancreaticoduodenectomy. The resection area includes the head of the pancreas, duodenum, gallbladder, and common bile duct (surrounded by black dotted lines). (**b**) Intraoperatively, pancreatic juice is collected after the pancreas is resected. A pancreatic duct tube is inserted into the stump of the pancreatic duct to collect pancreatic juice. (**c**) Reconstruction after pancreaticoduodenectomy is performed using Child’s modified method, with an external fistula tube placed in the pancreatic duct. On the seventh day after surgery, pancreatic juice is collected from the externalized pancreatic duct tube.

Pancreatic juice samples were collected intraoperatively and on postoperative day 7. Extent of resection in pancreaticoduodenectomy is shown in Figure 1a. During surgery, a tube was inserted into the pancreatic duct immediately after pancreatic transection, and expelled pancreatic juice was collected (Figure 1b). On postoperative day 7, pancreatic juice was collected from the external drainage tube (Figure 1c). To ensure that only pancreatic juice was collected, fluid was obtained exclusively from a tube that had been inserted into the pancreatic duct, allowing selective recovery of pancreatic juice and minimizing contamination. Collection was performed via a closed drainage system. Additionally, the drainage tube and collection devices were handled aseptically. Any fluid showing visible blood, bile, or turbidity suggestive of contamination, and cases in which the pancreatic duct tube had dislodged from the pancreatic duct, were excluded from analysis. Pancreatic juice was promptly stored on ice, measured in the laboratory, and then rapidly frozen in liquid nitrogen. After freezing, samples were stored at −80 °C for long-term preservation. Postoperative pancreatic juice samples were not collected from patients whose external pancreatic duct had fallen off by the seventh day after surgery.

### 2.4. Chemicals and Reagents

Methanol, acetonitrile, ultra-pure water, and formic acid of LC-MS grade were used in all experiments and were purchased from Wako Pure Chemical Industries (Osaka, Japan).

### 2.5. Sample Preparation

Pancreatic juice samples were stored at −80 °C and thawed immediately prior to use. Metabolites in pancreatic juice were extracted as follows. First, 20 µL of pancreatic juice was mixed with 80 µL of MeOH at room temperature, vigorously shaken, and allowed to stand for 30 min. After a second shaking, the mixture was centrifuged (14,000× *g*, 10 min, 4 °C) to obtain supernatant, which was analyzed using liquid chromatography quadrupole time-of-flight mass spectrometry (LC-QTOF-MS).

### 2.6. LC-QTOF-MS Analysis

LC-QTOF-MS analysis was performed using an LCMS-9030 system (Shimadzu, Kyoto, Japan) based on a previously reported method [36,37]. First, 1 or 2 µL of sample solution were injected into an LC-QTOF-MS system. An Accura Triart C_18_ (2.1 × 100 mm, 1.9 µm; YMC, Kyoto, Japan) was used for metabolite separation. Mobile phase A consisted of 0.1% (*v*/*v*) formic acid in ultra-pure water and mobile phase B consisted of 0.1% (*v*/*v*) formic acid in MeOH/acetonitrile (1:1, *v*/*v*). Metabolites were eluted from the column with the following gradient program at a flow rate of 0.32 mL/min. Starting at 0% B, initial conditions were maintained for 1 min, increased to 5% B between 1 and 3 min, increased to 90% B between 3 and 9 min, increased to 100% B between 9 and 11 min, maintained at 100% B from 11 to 15 min, returned to 0% B between 15 and 15.5 min, and maintained at 0% B from 15.5 to 20 min. The time required to measure one sample was 20 min. The column temperature was 45 °C and the sample cooler temperature was 4 °C.

Thereafter, 1 and 2 µL of sample were injected in positive (pos) and negative (neg) ion modes, respectively. Data acquisition and mass spectrometric analyses were performed according to our previously reported method [36]. Briefly, samples were analyzed separately in positive and negative ion modes using electrospray ionization over an m/z range of 70–900. Instrument calibration, quality control procedures, full-scan MS1 acquisition, and data-dependent MS/MS analyses were conducted as described previously. Relative abundances were evaluated based on MS1 parent ion intensities, and MS/MS spectra were obtained for structural determination. Extract of pancreatic juice from a representative patient with pancreatic cancer was used as a pooled quality control (QC) sample. The QC sample was injected five times (at the beginning, middle, and end of the batch) to evaluate detection stability throughout the run (Supplementary Table S3), confirming consistent peak detection during the entire sample analysis. All data were collected and processed using LabSolutions software version 5.118 for the LCMS-9030 (Shimadzu).

### 2.7. Data Processing and Annotation

Raw data (mzML format) were processed and compounds were annotated using MS-DIAL (ver. 5.1.230129) and MS-FINDER (ver. 3.56) [38,39]. Peak detection, alignment, integration, and identification were performed as described previously [36]. As necessary, MS1 ions were searched against the HMDB metabolite database (ver. 5.0), MassBank of North America, MetFrag, and the LIPID MAPS lipid database (all accessed on 14 January 2025). If MS2 spectra matched a well-known blood metabolite, this compound was annotated as Rank A. If a significant MS2 spectrum could not be obtained, but the MS1 value matched the only metabolite registered in HMDB, it was also annotated as Rank A. If MS1 and/or MS2 spectra matched, but could not be resolved to a single compound due to multiple candidate metabolite hits, a compound that we judged most likely to be a serum metabolite was annotated as Rank B.

### 2.8. Metabolite Selection and Model Construction

Multivariate analyses enabled clear discrimination between the two groups, revealing differences in metabolites between them. A partial least-squares discriminant analysis (PLS-DA) was performed with MetaboAnalyst version 6.0 (accessed on 14 January 2025). Characteristic metabolites were identified by comparing the pancreatic cancer group with the group of patients with other benign or malignant diseases, including chronic pancreatitis and non-pancreatic malignancies such as distal bile duct adenocarcinoma and ampullary adenocarcinoma. Non-targeted metabolomics and extraction of characteristic metabolites were performed as previously described [40,41,42]. Selection and refinement of metabolites were performed in a two-step, exploratory procedure (Figure 1). Over 10,000 peaks were detected by means of LC-QTOF-MS in both bile and serum metabolomic analyses. Metabolic features were subjected to partial least squares discriminant analysis (PLS-DA) using MetaboAnalyst 6.0 (accessed on 14 January 2025). To enhance the robustness of candidate selection, four independent PLS-DA models were constructed: positive-ion mode with autoscaling, positive-ion mode with Pareto scaling, negative-ion mode with autoscaling, and negative-ion mode with Pareto scaling. From each model, the top 30–40 features with the highest Variable Importance in Projection (VIP) scores were selected as preliminary candidate metabolites. After merging the four candidate lists, removing duplicate features detected under multiple ionization modes or scaling methods, and excluding obvious blank-derived or contaminant peaks by visual inspection, 56 metabolic features were retained as primary candidates for second-stage modeling. This VIP-based selection step was conducted as an exploratory dimensionality-reduction procedure and not as definitive biomarker identification. Because this feature-selection step was performed prior to subsequent model validation, the entire pipeline does not constitute a fully nested modeling framework, and potential optimistic bias in predictive performance cannot be completely excluded.

In the second stage, these candidate metabolites were subjected to machine-learning-based modeling. A binary prediction model discriminating between PDAC and non-PDAC was developed using least absolute shrinkage and selection operator (LASSO) logistic regression. Because of the limited sample size, no independent validation cohort was available. Therefore, internal validation was performed using 3-fold cross-validation (repeated three times; nine test sets in total) on the discovery cohort. Importantly, all cross-validation procedures were performed using only the final set of preselected metabolites (3 metabolites per model, derived from LASSO logistic regression following initial PLS-DA-based candidate screening). This approach avoids nested cross-validation and thus carries a risk of optimistic bias in performance estimates due to potential information leakage from the feature selection step. Model robustness was additionally evaluated via leave-one-out cross-validation (LOOCV), permutation testing, and .632+ bootstrap resampling. Complementary model interpretability was provided by SHAP (SHapley Additive exPlanations) analysis. All analyses were performed using R (ver. 4.4.2). It should be emphasized that internal cross-validation provides only an estimate of internal model consistency and does not represent external generalizability. Therefore, reported model performances should be interpreted as exploratory, and independent external validation will be required to establish any clinical or practical utility.

## 3. Results

### 3.1. Patient and Sample Characteristics

Four groups of pancreatic juice samples were analyzed: intraoperative pancreatic juice from patients with pancreatic cancer (Group A, PDAC, *n* = 11), intraoperative pancreatic juice from patients with other benign or malignant diseases, including chronic pancreatitis and non-pancreatic malignancies such as distal bile duct adenocarcinoma and ampullary adenocarcinoma (Group B, non-PDAC, *n* = 14), postoperative pancreatic juice derived from normal pancreatic tissue after pancreatic cancer resection (Group C, non-PDAC, *n* = 7), and postoperative pancreatic juice from non-PDAC cases (Group D, non-PDAC, *n* = 9). Table 1 shows the medical details of pancreatic cancer patients. Table 2 shows medical backgrounds of patients with non-pancreatic cancers. Because this group included a variety of pathological conditions, Supplementary Table S1 provides a reclassification of patients originally included in Table 2 (non-PDAC group). This supplementary table presents a case-by-case correspondence between each patient and the following categories: biliary/ampullary carcinoma, precancerous/inflammatory lesions, and others, e.g., duodenal adenomas. In addition, detailed clinical background and medication information relevant to the metabolomic analysis—including history of metabolic disorders, chronic alcohol consumption, use of medications potentially affecting the pancreas, and the presence or absence of preoperative chemotherapy—are provided in Supplementary Table S2. Postoperative pancreatic juice samples were not collected from patients whose external pancreatic ducts had fallen off by the seventh day after surgery. Pancreatic juice specimens exhibited heterogeneous physical appearances. Intraoperative samples often varied in degree of reddish color, turbidity, and viscosity, potentially reflecting contamination with blood, insoluble material, and/or polysaccharides. In contrast, postoperative pancreatic juice samples were generally colorless and non-viscous, although turbidity was variable. Such variability in sample properties may have contributed to the heterogeneity observed in metabolomic analysis.

### 3.2. Separation of Pancreatic Cancer and Non-Cancer Patients via Partial Least-Squares Discriminant Analysis

**Figure 2:**
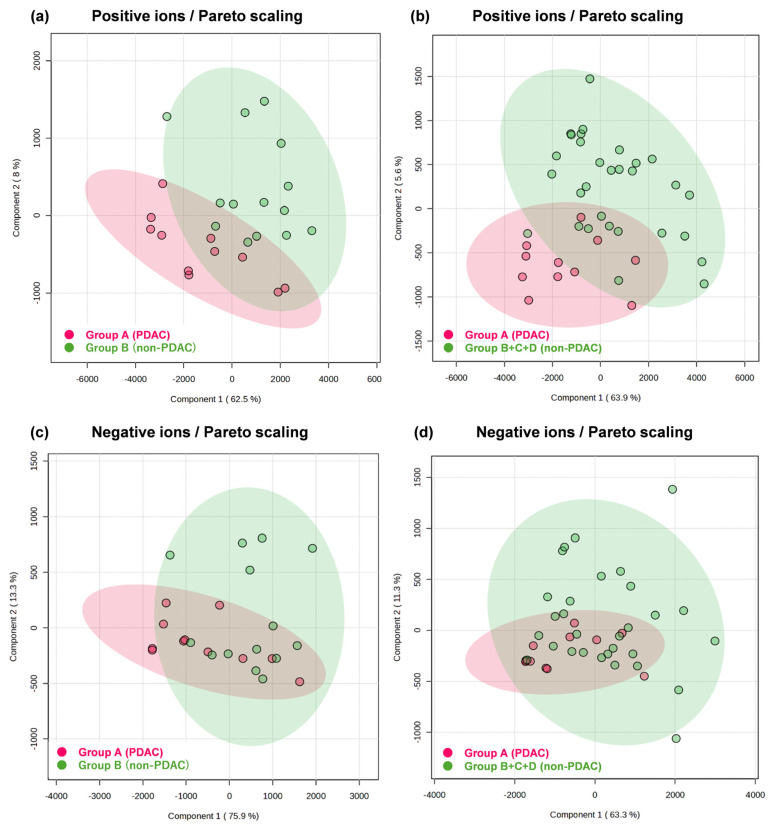
Partial Least-Squares Discriminant Analysis. PLS-DA of positive-ion-mode data comparing Group A with B (**a**) and with B + C + D (**b**), and PLS-DA of negative ion mode data comparing group A and B (**c**) and B + C + D (**d**). Group A, intraoperative pancreatic juice from PDAC patients; Group B, intraoperative pancreatic juice from non-PDAC patients; Group C, postoperative external drainage pancreatic juice from PDAC patients; Group D, postoperative external drainage pancreatic juice from non-PDAC patients.

Non-targeted metabolomic profiling of pancreatic juice samples was performed using LC-QTOF-MS. After alignment and quality filtering with MS-DIAL, several hundred ion peaks were subjected to statistical analysis. Both positive and negative ion datasets were analyzed using partial least-squares discriminant analysis (PLS-DA) in MetaboAnalyst. In PLS-DA plots comparing the PDAC group with the non-PDAC group, partial but distinct separation was observed, indicating clear differences in overall metabolomic profiles between PDAC and non-PDAC pancreatic juice. Positive ion mode (Figure 2a) showed clearer clustering than negative ion mode (Figure 2c), suggesting that lipid-related metabolites contributed strongly to group discrimination. When the PDAC group was compared with the combined non-PDAC group, a similar tendency was observed, with partial separation between the two clusters (Figure 2b,d). Although overlap remained due to biological and sampling variability, PLS-DA results consistently suggested that PDAC pancreatic juice contains metabolite patterns distinct from those of non-PDAC pancreatic juice.

### 3.3. Exploration of Potential Marker Metabolites

Fifty-six metabolites were selected as important contributors to group discrimination based on VIP scores. Of these, 19 features were annotated as Rank A and 10 putative candidate metabolites (marked with Δ) were annotated as Rank B. Individual properties of these 56 metabolites, including unknown compounds, are listed in Table 3. Compound annotations were performed based on MS/MS spectra using MS-DIAL (ver. 5.1), MS-FINDER (ver. 3.56), and publicly available metabolite databases such as HMDB. Given the high variability in postoperative pancreatic juice collected through the external drainage tube, only intraoperative pancreatic juice (A vs. B) was used for further comparisons. The annotated metabolites are shown in Supplementary Figure S2.

### 3.4. Metabolites Altered in PDAC Pancreatic Juice

Several metabolites showed a decreasing trend in PDAC samples. Citric acid and isocitric acid, both intermediates of the TCA cycle, showed a decreasing trend in PDAC compared with non-PDAC. Their mean peak intensities were reduced by approximately 40–50% in PDAC pancreatic juice. Similarly, oxidized fatty acids, including stearic acid oxide [FA(18:0)+2O] and linoleic acid oxide [FA(18:2)+2O], showed a decreasing trend in PDAC compared with non-PDAC. These oxidized fatty acids are primarily generated through lipid peroxidation and oxidative stress. Putative guanosine and N-oleoyl glycine also showed a decreasing trend in pancreatic juice derived from pancreatic cancer.

Lactate, a glycolytic end product often elevated in tumor-associated fluid, showed no significant differences between PDAC and non-PDAC. This result contrasts with previous reports that lactate accumulates in PDAC pancreatic juice, suggesting the influence of sample handling and individual differences [43].

### 3.5. Development of a Pancreatic Juice Metabolomic Model for Diagnosing Pancreatic Cancer

**Figure 3:**
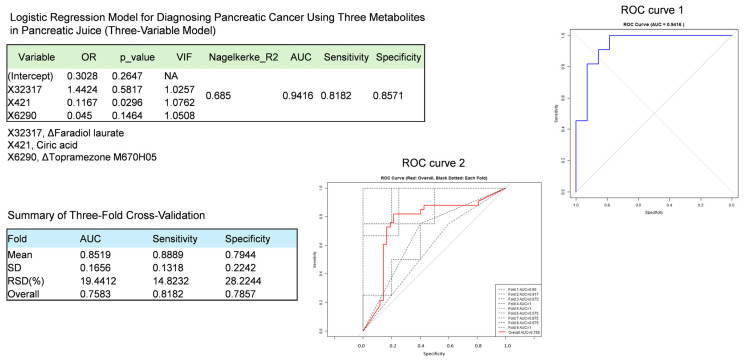
Three-Variable Model for Pancreatic Cancer Diagnosis Using Pancreatic Juice Data on 56 Metabolites. Model performance was optimized using LASSO logistic regression to select three variables from 56 metabolic features. Performance of the resulting three-variable model with its ROC curve (ROC Curve 1), based on data from all samples. Metabolomic data were obtained from pancreatic juice samples collected from patients. Model performance was validated using three-fold cross-validation on the discovery cohort. Nine ROC curves generated from three iterations of three-fold cross-validation are shown, as well as the mean and variability of AUC, sensitivity, and specificity. These ROC curves are depicted as black dotted lines (ROC Curve 2), while the overall prediction ROC curve is shown as a solid red line. Because feature selection was performed outside the cross-validation framework—with CV applied only to the final model—the cross-validated performance is likely subject to optimistic bias due to data leakage. Model construction and validation were performed using R (version 4.4.2).

In a retrospective discovery cohort, non-targeted metabolomic profiling was performed on preserved pancreatic juice samples collected from patients with PDAC and non-PDAC controls, as described previously. A PLS-DA approach was employed to detect metabolic features that discriminated between the two groups, resulting in the selection of 56 candidate features from pancreatic juice samples. These 56 metabolites were then subjected to LASSO logistic regression to construct a binary classification model for PDAC diagnosis. A high-performing model comprising three metabolic features, X421 (citric acid), X32317, and X6290, achieved the best discrimination (Figure 3). In this three-feature model trained on intraoperative pancreatic juice samples, the receiver operating characteristic (ROC) curve yielded an area under the curve (AUC) of 0.942, with sensitivity of 0.818 and specificity of 0.857 in distinguishing PDAC (*n* = 11) from non-PDAC (*n* = 14) patients. Violin plots for pancreatic juice metabolites included in the logistic regression models are shown in Supplementary Figure S3.

To mitigate overfitting and to evaluate model performance, 3-fold cross-validation repeated three times was performed using the discovery cohort. The mean (±SD) performance among the nine resulting ROC curves was an AUC of 0.852 ± 0.166, sensitivity of 0.889 ± 0.132, and specificity of 0.794 ± 0.224 (Figure 3). When evaluated on pooled cross-validation predictions, the model yielded an overall AUC of 0.758, sensitivity of 0.818, and specificity of 0.786. Although these cross-validated metrics were modestly lower than those obtained from the model trained on the entire discovery dataset, the model retained reasonable discriminatory ability. In addition, LOOCV (*n* = 25), permutation testing (*n* = 1000) and bootstrap resampling (*n* = 1000) were performed for the final three-variable model to assess the likelihood of chance performance and internal stability. The model achieved a robust AUC of 0.805 via LOOCV (Supplementary Figure S1). The permutation test yielded an observed AUC of 0.942 with an empirical *p*-value of 0.003, and the “.632+ bootstrap” analysis showed a 95% confidence interval of 0.773–0.948 for the AUC (Supplementary Figure S1). SHAP analysis showed that model predictions were driven by a limited number of key metabolites, with variable contribution patterns across individuals (Supplementary Figure S2). These findings indicate that the three-feature metabolomic model has potential clinical utility as a diagnostic tool for pancreatic cancer, pending confirmation in independent validation cohorts.

Given that two of the three features in the initial model were metabolites with only Rank B confidence annotations, we next sought to construct a diagnostic model using exclusively Rank A-annotated metabolites. The best-performing model again comprised three metabolites: X421 (citric acid), X11488 (oleoylglycerol), and X1515 (FA(18:2)+2O) (Supplementary Figure S3). When trained on intraoperative pancreatic juice samples, this second three-feature model achieved an AUC of 0.870, with sensitivity of 0.636 and specificity of 0.756. The same 3-fold cross-validation procedure (repeated three times) was applied. Across the nine samples, the model showed a mean (±SD) AUC of 0.782 ± 0.112, sensitivity of 0.787 ± 0.177, and specificity of 0.811 ± 0.188. Pooled cross-validation predictions yielded an overall AUC of 0.753, sensitivity of 0.576, and specificity of 0.857 (Supplementary Figure S3). Although this Rank A-only model retained moderate discriminatory ability, its performance was clearly inferior to that of the original three-feature model that included Rank B metabolites.

## 4. Discussion

The present non-targeted metabolomic analysis of pancreatic juice demonstrated metabolic alterations associated with pancreatic ductal adenocarcinoma (PDAC). The clear, though partial, separation between the PDAC and non-PDAC groups in multivariate analyses suggests that pancreatic juice reflects disease-specific metabolic profiles. Because pancreatic juice originates from pancreatic ductal epithelial cells, it can sensitively capture metabolic changes occurring in tumor-adjacent tissue. This indicates that pancreatic juice metabolomics is a biologically meaningful and potentially valuable approach for biomarker discovery and for revealing metabolic reprogramming in PDAC.

Intermediates of the tricarboxylic acid (TCA) cycle, such as citric acid and isocitric acid, tended to decrease in PDAC pancreatic juice. Similarly, oxidized fatty acids derived from linoleic, oleic, and linolenic acids also tended to decrease. These findings are consistent with metabolic features of the Warburg effect, in which cancer cells favor aerobic glycolysis over mitochondrial oxidative phosphorylation [27,44,45]. Downregulation of TCA intermediates suggests suppression of mitochondrial function [46] and a metabolic shift toward glycolysis [47,48], which provides biosynthetic precursors necessary for rapid cell proliferation [49]. The decrease in oxidized fatty acids may also indicate attenuated β-oxidation of fatty acids under hypoxic conditions in the PDAC microenvironment, resulting in reduced oxidative metabolism. Previous studies have reported that mitochondrial metabolism is suppressed in PDAC cells due to oncogenic KRAS signaling and altered redox regulation [50,51,52], further supporting our observations that tumor metabolic adaptation is reflected in pancreatic juice composition.

Unlike previous reports, this study did not detect a significant increase in lactate concentration in PDAC pancreatic juice [18,28]. The absence of a clear difference in lactate may be explained by variability in sample collection and handling. Intraoperative pancreatic juice samples, which were used in this study, may differ in composition from postoperative or endoscopic samples analyzed in earlier studies. Factors such as minor blood contamination, variation in viscosity or turbidity, and differences in preservation time on ice may all contribute to lactate instability. Furthermore, lactate production and secretion can vary depending on tumor volume, stromal content, and local oxygen tension. These methodological and biological differences could account for the inconsistency with previous findings.

In addition, the logistic regression model constructed using the three significantly altered metabolites differentiated PDAC from non-PDAC cases to a large degree. ROC analysis demonstrated moderate discriminative performance of the model, suggesting that these metabolites may have utility for diagnosis of pancreatic cancer. Importantly, our metabolomic signature (AUC 0.852, sensitivity 0.889, and specificity 0.794) shows sensitivity that is comparable or superior to previously reported pancreatic juice biomarkers, including KRAS cfDNA [16,53], P53 cfDNA [16,53], methylated DNA fragments [53], and microRNAs [20], with specificity also at a similar level. Notably, since our signature reflects metabolic pathways distinct from those captured by DNA- or RNA-based markers [15,16,17,18,19,20], it may provide complementary diagnostic information, and combination with existing biomarkers could further improve accuracy. In this context, integrative approaches combining metabolomic signatures with previously reported protein biomarkers in pancreatic juice, as well as with blood-based markers, may enhance diagnostic performance and represent an important direction for future multi-omics studies. These findings are consistent with the growing evidence from liquid biopsy approaches, including analysis of extracellular vesicles such as exosomes [15], circulating nucleic acids [16,17,18,19], tumor-associated proteins [21,22,23], and lipids [24], which provide comprehensive molecular tumor profiles and are increasingly applied in clinical cancer diagnostics [25,26,27,28,29,30]. Although preliminary, these findings indicate that metabolomic signatures derived from pancreatic juice could support future diagnostic approaches, pending further validation in independent cohorts due to the exploratory and small-scale nature of this study.

Notably, citric acid was consistently selected across models and showed a decreasing trend in PDAC. As discussed above, this reduction is compatible with hypoxia-related metabolic reprogramming in cancer. Furthermore, in an additional model restricted to Rank A annotations, an oxidized fatty acid (FA(18:2)+2O) and monoacylglycerol (oleoylglycerol) were also selected and tended to be lower in PDAC.

Several limitations are acknowledged. First, this was an exploratory pilot study with a relatively small discovery cohort (PDAC *n* = 11, non-PDAC *n* = 14), which raises concerns regarding statistical power, potential overfitting, and limited generalizability. Because feature selection and model construction were performed using the same dataset, some degree of optimistic bias in predictive performance cannot be entirely excluded. To further assess model robustness beyond 3-fold cross-validation, additional validation procedures were performed, including leave-one-out cross-validation (LOOCV), permutation testing, and .632+ bootstrap resampling. These supplementary analyses yielded performance estimates consistent with the primary model, supporting relative model stability within this exploratory dataset. However, given the limited sample size, these internal validation approaches cannot substitute for independent external validation. Therefore, the present findings should be considered hypothesis-generating. They require validation in larger, independent cohorts. Second, the non-PDAC group was intentionally heterogeneous and included biliary and ampullary carcinomas, precancerous lesions such as intraductal papillary mucinous neoplasms and intraductal papillary mucinous adenomas, and inflammatory conditions including chronic pancreatitis. While this design reflects real-world diagnostic scenarios in which PDAC must be differentiated from clinically relevant mimics, it may have introduced biological heterogeneity and confounding effects that attenuated group separation in multivariate analyses. Future studies with larger sample sizes will enable stratified analyses separating malignant biliary-ampullary tumors from precancerous or inflammatory lesions to further refine PDAC-specific metabolic signatures. Third, most of the 56 PLS-DA–selected features were unannotated or assigned Rank B confidence, and metabolite identification therefore remains incomplete. Finally, although LASSO logistic regression was used to derive an exploratory three-metabolite model, this model should be regarded as hypothesis-generating and requires independent validation in larger, prospectively collected cohorts with more strictly stratified comparison groups. In addition, pancreatic juice collection is inherently invasive and may be influenced by procedural factors, variability in sample volume and composition, and differences in collection protocols. These factors may limit direct comparison across exploratory studies and should be carefully standardized in future investigations.

Taken together, metabolomic changes observed in this study, namely, reduced TCA cycle intermediates and oxidized fatty acids along with increased complex lipids, are consistent with a metabolic shift in PDAC that may reflect suppressed oxidative metabolism under hypoxic conditions, accompanied by relative enhancement of lipid biosynthetic pathways. While these alterations do not directly demonstrate metabolic flux, they suggest that pancreatic juice metabolomics can capture aspects of tumor-associated metabolic reprogramming. These findings may provide a foundation for future studies aimed at evaluating pancreatic juice–derived metabolites as candidate biomarkers.

## 5. Conclusions

In conclusion, non-targeted LC-QTOF-MS analysis of pancreatic juice suggests that PDAC is associated with altered energy metabolism, characterized by reduced levels of TCA cycle intermediates and oxidized fatty acids, potentially reflecting a metabolic shift toward glycolysis. In this exploratory pilot study, a diagnostic model comprising three selected metabolites demonstrated moderate discriminative performance for differentiating PDAC from non-PDAC cases. These findings indicate that pancreatic juice metabolomics may capture tumor-associated biochemical alterations with potential for future biomarker development. However, given the limited sample size and the lack of independent external validation, the present results should be interpreted as tentative. Further large-scale, prospective, and reproducible studies are required to confirm the diagnostic utility, robustness, and biological significance of these metabolic signatures before any clinical application can be considered.

## Figures and Tables

**Table 1:** Background and histological diagnoses of patients with invasive pancreatic ductal adenocarcinoma.

No.	Sex	Age	Histology	Tumor Location	Stage
1	female	60	Moderately differentiated ductal adenocarcinoma	Head of pancreas	pT3N1aM0
2	female	79	Moderately differentiated ductal adenocarcinoma	Head of pancreas	pT3N1aM0
3	male	83	Well-differentiated ductal adenocarcinoma	Head of pancreas	pT3N0M0
4	male	73	Moderately differentiated ductal adenocarcinoma	Head of pancreas	pT3N1aM0
5	male	73	Well-differentiated ductal adenocarcinoma	Body of pancreas	pT1N0M0
6	female	63	Well-differentiated ductal adenocarcinoma	Head of pancreas	pT3N1aM0
7	female	48	Moderately differentiated ductal adenocarcinoma	Head of pancreas	pT3N2M0
8	male	68	Well-differentiated ductal adenocarcinoma	Head of pancreas	pT3N1bM0
9	female	76	Poorly differentiated ductal adenocarcinoma	Head of pancreas	pT3N0M0
10	male	69	Well-differentiated ductal adenocarcinoma	Head of pancreas	pT3N1aM0
11	male	70	Well-differentiated ductal adenocarcinoma	Head of pancreas	pT3N1aM0

**Table 2:** Background and histological diagnoses of patients with non-invasive pancreatic ductal carcinoma.

No.	Sex	Age	Histology
1	female	67	Lower bile duct carcinoma
2	male	61	Duodenal neuroendocrine tumor
3	male	76	Duodenal adenoma
4	male	75	Ampullary adenoma
5	female	82	Ampullary adenocarcinoma
6	female	70	Intraductal papillary mucinous carcinoma
7	male	66	Chronic pancreatitis
8	female	70	Intra-ampullary papillary-tubular neoplasm
9	male	70	Intraductal papillary mucinous carcinoma
10	male	72	Intraductal papillary mucinous carcinoma
11	male	77	Lower bile duct carcinoma
12	male	67	Intraductal papillary mucinous carcinoma
13	male	65	Duodenal adenocarcinoma
14	male	77	Intraductal papillary mucinous carcinoma

**Table 3:** Individual Characteristics of 56 Candidate Metabolites Selected by means of PLS-DA.

Peak ID	RT	m/z	Annotation	Adduct Type	Group A/B		Group A/BCD	
					FC	*p*-Value	FC	*p*-Value
1505	0.775	173.0248	UK	[M+H]+	0.3720	0.0014	0.7477	0.3951
680	0.844	140.0712	UK	[M+H]+	1.4517	0.0970	2.5220	0.0020
1034	0.846	156.0452	UK	[M+H]+	1.5778	0.0525	2.4611	0.0013
624	0.866	137.071	UK	[M+H]+	1.4598	0.1327	2.1465	0.0070
4325	0.943	446.1559	UK	[M+FA-H]-	0.1382	0.2870	0.0969	0.0056
5132	0.946	468.1363	UK	[M-H]-	0.2799	0.2943	0.1257	0.0011
422	1.541	191.0209	Isocitric acid	[M-H]-	0.4801	0.0233	0.6878	0.1699
421	1.857	191.0208	Citric acid	[M-H]-	0.5426	0.0057	0.8482	0.4501
1868	5.036	330.0714	UK	[M-H]-	1.6602	0.2932	2.9933	0.0796
6295	5.049	284.1031	ΔGuanosine	[M+H]+	0.4179	0.0043	0.5495	0.0600
2578	5.158	373.1752	ΔLeu-Asp-Gln	[M-H]-	0.1446	0.2527	0.1040	0.0058
3008	5.191	394.1647	UK	[M-H]-	0.2511	0.1942	0.2105	0.0270
4126	5.286	241.075	UK	[M+H]+	1.6597	0.0769	3.4425	0.0005
2079	5.543	343.2012	FA(18:2)+4O	[M-H]-	0.5954	0.2064	0.1693	0.0282
5410	5.892	268.0667	UK	[M+H]+	0.0000	0.0363	0.0000	0.0013
13090	6.2	377.2189	UK	[M+H]+	1.4922	0.1615	3.1094	0.0047
2359	6.453	361.225	UK	[M-H]-	0.4383	0.0145	0.3113	0.0085
6290	6.483	284.0624	ΔTopramezone M670H05	[M+H]+	0.0575	0.0323	0.0120	0.0001
17249	7.393	427.2752	UK	[M+H]+	0.2019	0.0104	0.2553	0.0044
10491	7.489	344.4856	UK	[M+H]+	0.4473	0.0111	0.5805	0.0655
10468	7.492	344.2595	ΔDodecanoylcarnitine	[M+H]+	0.6509	0.0839	0.6773	0.0872
10571	7.493	345.2541	UK	[M+H]+	0.5680	0.0482	0.6115	0.0830
1756	7.498	325.2054	UK	[M-H]-	0.5474	0.0563	0.5760	0.0828
1838	7.569	329.2356	FA(18:1)+3O	[M-H]-	0.5485	0.0761	0.5141	0.0457
1837	7.812	329.2326	FA(18:1)+3O	[M-H]-	0.5349	0.0720	0.4980	0.0389
9164	7.87	328.2513	UK	[M+H]+	0.5316	0.0977	0.5468	0.1033
1576	8.076	313.2408	ΔFA(18:1)+2O	[M-H]-	0.5299	0.0996	0.4760	0.0700
2150	8.127	347.2018	UK	[M-H]-	0.5511	0.1485	0.2862	0.0275
7011	8.154	295.2305	ΔFA(18:3)+O	[M+H]+	0.6536	0.0970	0.6227	0.0368
7069	8.156	295.7082	UK	[M+H]+	0.4805	0.0093	0.5370	0.0268
8183	8.157	313.2458	ΔFA(18:2)+2O	[M+H]+	0.6272	0.0418	0.6516	0.0523
1515	8.155	311.2277	FA(18:2)+2O	[M-H]-	0.5693	0.0318	0.6173	0.0628
9783	8.161	335.2266	UK	[M+H]+	0.5663	0.0354	0.5905	0.0449
1999	8.161	339.2021	UK	[M-H]-	0.6204	0.0197	0.6560	0.0378
7432	8.168	550.2121	UK	[M-H]-	0.5427	0.0104	0.6486	0.0722
35545	8.169	683.3995	UK	[M+H]+	0.5626	0.0222	0.6404	0.0729
8191	8.179	313.2801	UK	[M+H]+	0.3820	0.0120	0.5463	0.1152
2106	8.205	345.1346	UK	[M-H]-	0.5741	0.0661	0.5245	0.0226
3680	8.205	423.0779	UK	[M+FA-H]-	0.5661	0.0329	0.4316	0.0016
1044	8.209	277.1426	Phthalic acid ester (ΔMonoethylhexyl phthalic acid)	[M-H]-	0.5727	0.0641	0.5550	0.0371
1616	8.292	315.2583	FA(18:0)+2O	[M-H]-	0.5622	0.0297	0.6318	0.0888
9603	9.125	678.4218	UK	[M-H]-	0.4652	0.0853	0.7516	0.5449
10141	9.13	340.2938	ΔN-Oleoyl glycine	[M+H]+	0.5428	0.0421	0.7197	0.2875
11488	9.883	357.308	Oleoylglycerol	[M+H]+	0.1993	0.0972	0.3702	0.1648
39423	9.886	766.6536	UK	[M+H]+	0.0781	0.0901	0.1402	0.0813
18034	10.302	435.3422	UK	[M+H]+	0.6508	0.0063	0.7803	0.0361
9204	10.404	646.4316	UK	[M-H]-	0.4213	0.0552	0.8144	0.6251
10291	13.013	744.5681	UK	[M-H]-	1.7614	0.1884	3.1108	0.0368
43366	13.263	858.5104	UK	[M+H]+	1.6995	0.1124	3.0195	0.0131
14854	13.266	398.771	UK	[M+H]+	1.8275	0.1276	3.1335	0.0223
39051	13.274	759.599	ΔSM(d18:1/20:0)	[M+H]+	3.0388	0.0928	4.3587	0.0518
44037	13.323	884.5222	UK	[M+H]+	2.4662	0.1622	4.3915	0.0728
37639	13.95	726.559	UK	[M+H]+	2.7528	0.1187	5.8796	0.0470
30913	14.188	603.5464	UK	[M+H]+	2.5650	0.1285	4.7288	0.0480
32317	14.727	625.5503	ΔFaradiol laurate	[M+H]+	1.2766	0.0741	1.7054	0.0006
27576	16.01	551.5134	UK	[M+H]+	1.3933	0.0158	1.9041	0.0000

Δ denotes metabolites annoted as Rank B. Unkown peaks are labeled UK. *p*-values (from univariate Welch’s *t*-test) shown are uncorrected for multiple testing. After applying Benjamini–Hochberg false discovery rate (FDR) correction, no metabolites reached statistical significance (q < 0.05). Group A, intraoperative pancreatic juice from PDAC patients; Group B, intraoperative pancreatic juice from non-PDAC patients; Group C, postoperative external drainage pancreatic juice from PDAC patients; Group D, postoperative external drainage pancreatic juice from non-PDAC patients; FC, fold change; FA, fatty acid; SM, sphingomyelin.

## Data Availability

The data presented in this study are available upon request from the corresponding author.

## Supplementary Materials

The following supporting information can be downloaded at https://www.mdpi.com/article/10.3390/cancers18040684/s1, Table S1: Reclassification of patients originally included in Table 2 (non-PDAC group); Table S2: Demographic and clinical characteristics of study participants; Table S3: Reproducibility of detection peaks for representative metabolites in QC samples; Figure S1: Leave-One-Out, Permutation, and “.632+ Bootstrap” Validation of the Three-Variable Logistic Regression Model; Figure S2: SHAP-Based Feature Importance and Contribution Structure of the Three-Variable Logistic Regression Model; Figure S3: Three-Variable Model for Pancreatic Cancer Diagnosis using Pancreatic Juice Metabolomics Data of Rank A-Annotated Metabolites; Figure S4: Compound Annotations for Pancreatic Juice Metabolites in the Logistic Regression Models; Figure S5: Violin Plots for Pancreatic Juice Metabolites in the Logistic Regression Models.
