# Supplementary material for: Diagnostic Biomarkers for Pancreatic Ductal Adenocarcinoma Using Non-Targeted Metabolomic Analysis
Source: Cancers (Basel). 2026 Feb 19;18(4):684. doi: 10.3390/cancers18040684 (PMC12939614; doi:10.3390/cancers18040684)
Supplement: Supplementary file 1 [file cancers-18-00684-s001.zip › cancers-4139856-supplementary.pdf]

Supplementary Table S1

Supplementary Table S1. Reclassification of patients originally included in Table 2 (non-PDAC group).This table presents a case-by-case correspondence between each patient listed in Table 2 (non-PDAC group) and the following categories: biliary/ampullary carcinoma, precancerous/inflammatory lesions, and others (e.g., duodenal adenomas).

Case No.	Original histological diagnosis (Table 2)	Reclassified group
1	Lower bile duct carcinoma	Biliary / ampullary carcinoma
2	Duodenal neuroendocrine tumor	Others
3	Duodenal adenoma	Others
4	Ampullary adenoma	Biliary / ampullary carcinoma
5	Ampullary adenocarcinoma	Biliary / ampullary carcinoma
6	Intraductal papillary mucinous carcinoma	Precancerous / inflammatory
7	Chronic pancreatitis	Precancerous / inflammatory
8	Intra-ampullary papillary-tubular neoplasm	Biliary / ampullary carcinoma
9	Intraductal papillary mucinous carcinoma	Precancerous / inflammatory
10	Intraductal papillary mucinous carcinoma	Precancerous / inflammatory
11	Lower bile duct carcinoma	Biliary / ampullary carcinoma
12	Intraductal papillary mucinous carcinoma	Precancerous / inflammatory
13	Duodenal adenocarcinoma	Others
14	Intraductal papillary mucinous carcinoma	Precancerous / inflammatory

Supplementary Table S2

Supplementary Table S2. Demographic and Clinical Characteristics of Study Participants, Including Medical History, Medication Use, and Preoperative Treatments
Abbreviations: DM, diabetes mellitus; HT, hypertension; DL, dyslipidemia.

No. of samples	Sex	Age	Histology	Tumor location	Stage	Preoperative chemotherapy	Metabolic disorders	Chronic alcohol use	Medications affecting pancreas
**PDAC**									
1	female	60	Moderately differentiated ductal adenocarcinoma	Head of pancreas	pT3N1aM0	Yes	None	No	Gemcitabine
2	female	79	Moderately differentiated ductal adenocarcinoma	Head of pancreas	pT3N1aM0	No	DM, HT	No	None
3	male	83	Well-differentiated ductal adenocarcinoma	Head of pancreas	pT3N0M0	No	HT	Yes	None
4	male	73	Moderately differentiated ductal adenocarcinoma	Head of pancreas	pT3N1aM0	No	DM, HT	No	None
5	male	73	Well-differentiated ductal adenocarcinoma	Body of pancreas	pT1N0M0	No	DM, HT	No	None
6	female	63	Well-differentiated ductal adenocarcinoma	Head of pancreas	pT3N1aM0	Yes	HT	No	Gemcitabine
7	female	48	Moderately differentiated ductal adenocarcinoma	Head of pancreas	pT3N2M0	Yes	None	No	Gemcitabine
8	male	68	Well-differentiated ductal adenocarcinoma	Head of pancreas	pT3N1bM0	Yes	DM, HT	No	Gemcitabine, Metformin
9	female	76	Poorly differentiated ductal adenocarcinoma	Head of pancreas	pT3N0M0	Yes	DL	No	Gemcitabine
10	male	69	Well-differentiated ductal adenocarcinoma	Head of pancreas	pT3N1aM0	Yes	None	Yes	Gemcitabine
11	male	70	Well-differentiated ductal adenocarcinoma	Head of pancreas	pT3N1aM0	Yes	DL	No	Gemcitabine
**non-PDAC**									
1	female	67	Lower bile duct carcinoma			No	None	No	Steroids
2	male	61	Duodenal neuroendocrine tumor			No	HT	No	None
3	male	76	Duodenal adenoma			No	HT	No	None
4	male	75	Ampullary adenoma			No	DM, HT, DL	No	None
5	female	82	Ampullary adenocarcinoma			No	HT, DL	No	None
6	female	70	Intraductal papillary mucinous carcinoma			No	HT, DL	No	Steroids
7	male	66	Chronic pancreatitis			No	None	Yes	None
8	female	70	Intra-ampullary papillary-tubular neoplasm			No	None	No	Steroids
9	male	70	Intraductal papillary mucinous carcinoma			No	HT	No	None
10	male	72	Intraductal papillary mucinous carcinoma			No	HT	Yes	None
11	male	77	Lower bile duct carcinoma			No	None	No	None
12	male	67	Intraductal papillary mucinous carcinoma			No	None	Yes	None
13	male	65	Duodenal adenocarcinoma			No	HT	No	None
14	male	77	Intraductal papillary mucinous carcinoma			No	HT	No	None

Supplementary Table S3

Supplementary Table S3. Reproducibility of detection peaks for representative metabolites in QC samples

LC mode	Ion	Compounds	Adduct	m/z	RT(min)	Peak Intensities RSD(%) (n=5)
C18	pos	Choline	[M+H]+	104.109	0.804	11.660
		Inosine	[M+H]+	269.092	5.041	7.032
		PC(34:1)	[M+Na]+	782.583	13.966	10.564
	neg	Lactic acid	[M-H]-	89.025	1.483	4.899
		Uric acid	[M-H]-	167.022	2.279	8.247
		LPC(16:0)	[M+FA-H]-	540.331	8.988	1.333

Supplementary Figure S1

(A)

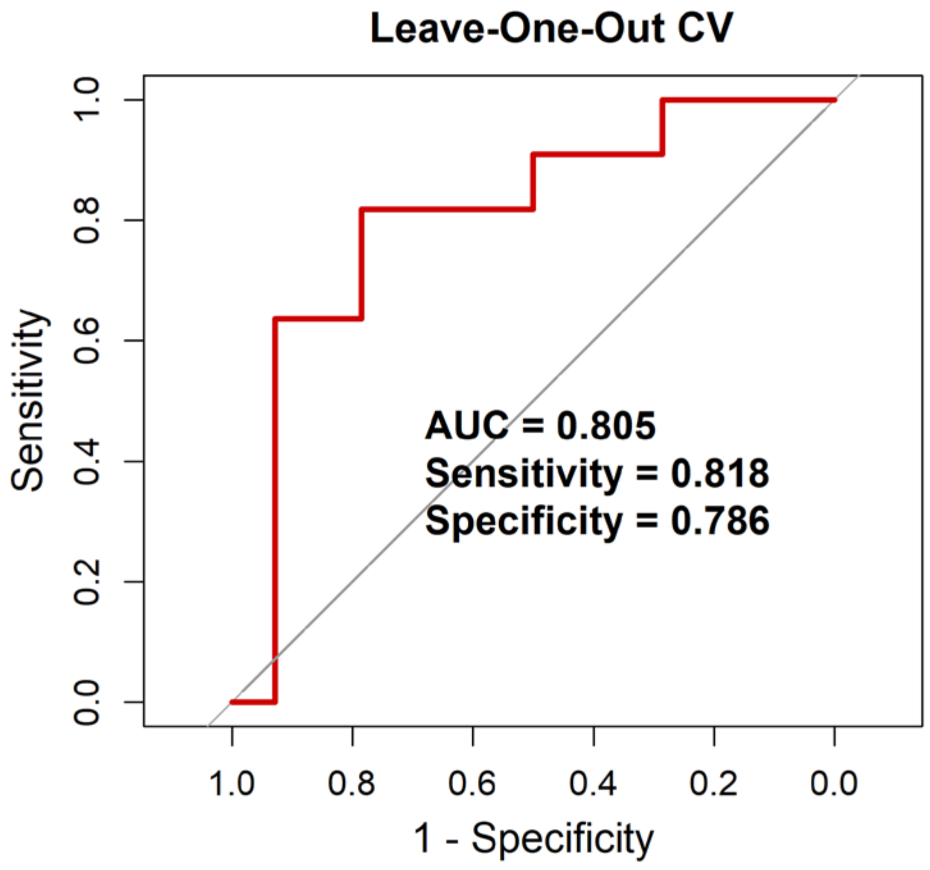

Supplementary Figure S1. Leave-One-Out, Permutation and “.632+ Bootstrap” Validation of the Three-Variable Logistic Regression Model

(A) Leave-One-Out cross-validation yielding AUC of 0.805 (n = 25). The ROC curve is indicated by red line. (B) Permutation test with 1,000 iterations showing the null distribution of AUC values under random class labels. The observed AUC (0.942) is indicated by the vertical red line, yielding an empirical p value of 0.003.(C) “.632+ Bootstrap” with 1,000 iterations showing the distribution of AUC values. The 95% confidence interval for the AUC was 0.773–0.948, indicating moderate internal stability of the model. Because feature selection and model validation were not fully nested within the cross-validation framework, the cross-validated performance may be subject to optimistic bias. Model building and validation were performed using R (ver. 4.4.2).

Supplementary Figure S1

(B)

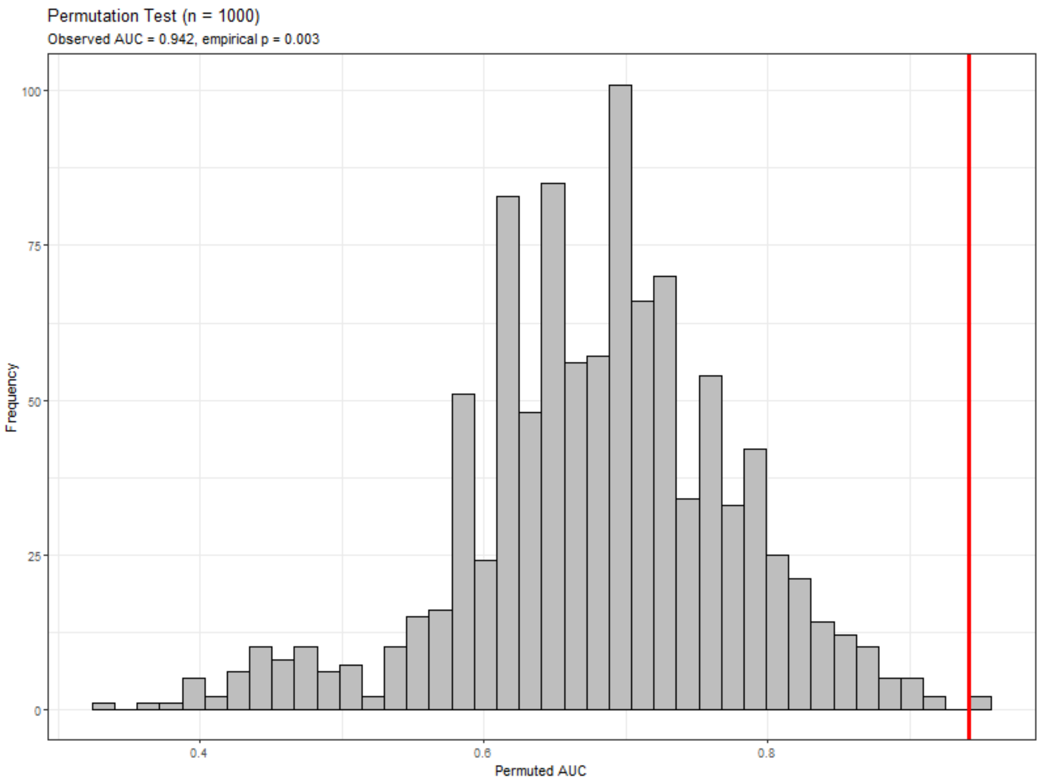

(C)

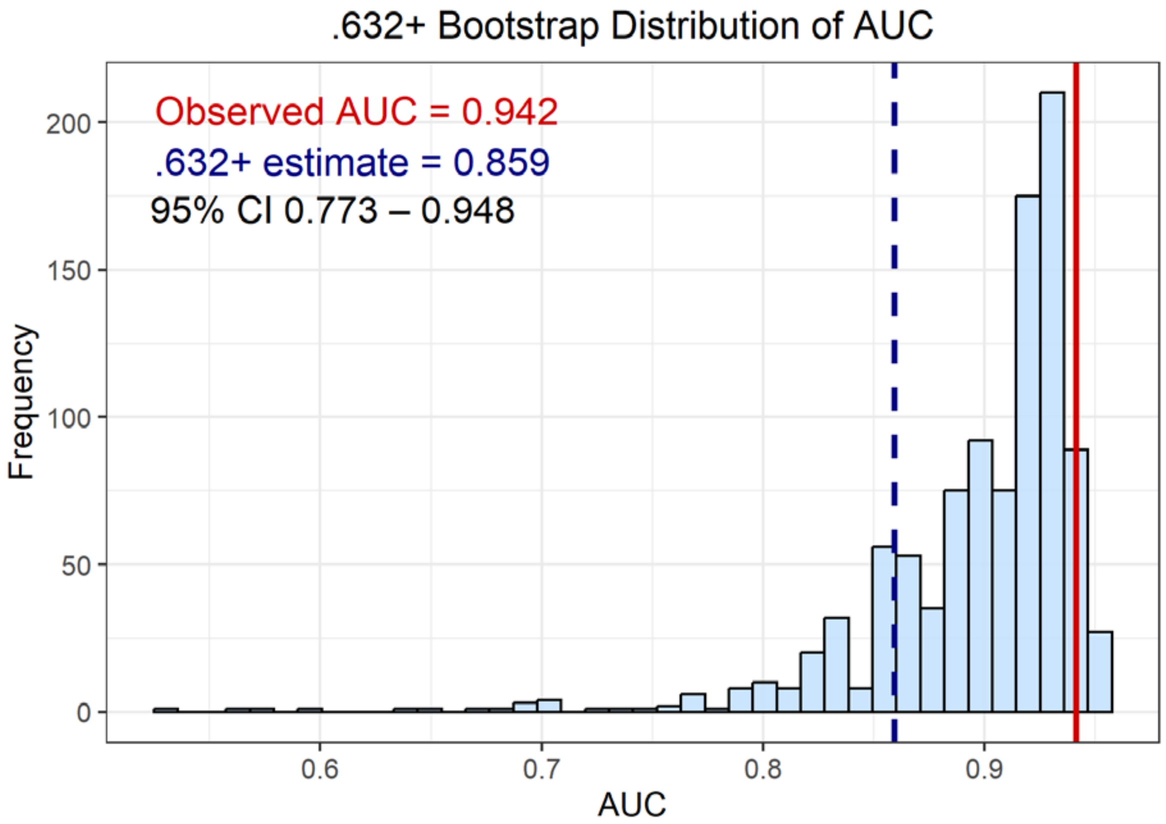

Supplementary Figure S2

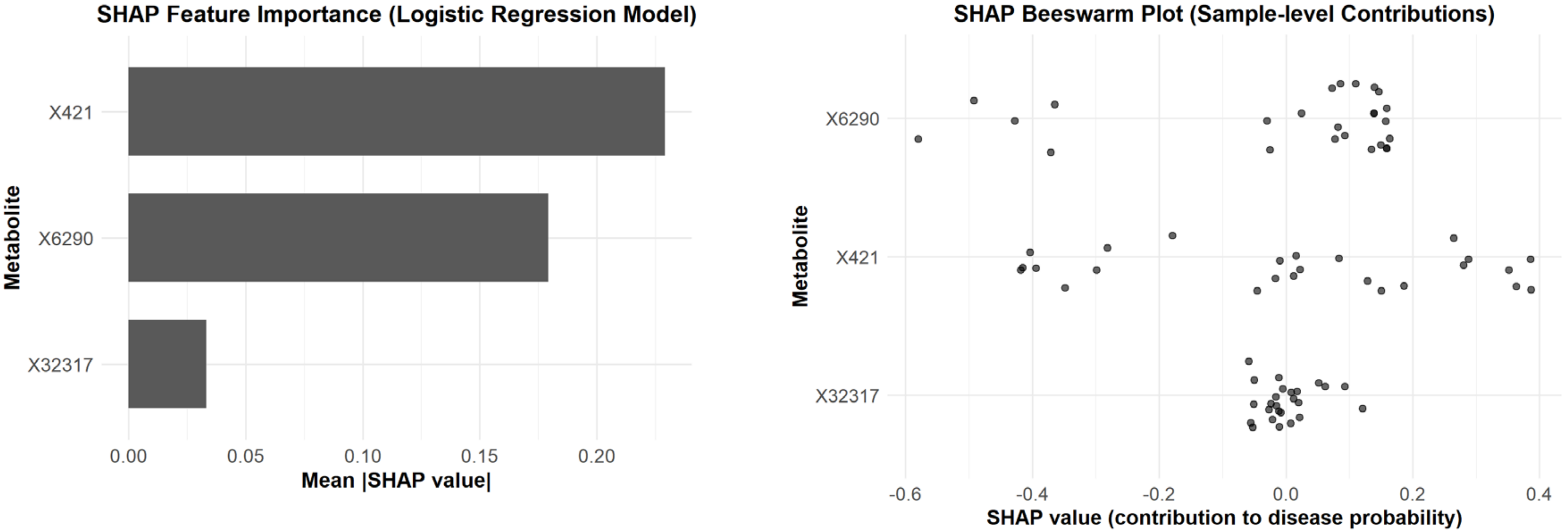

Supplementary Figure S2. SHAP-Based Feature Importance and Contribution Structure of Three-Variable Logistic Regression Model

SHAP analysis of the logistic regression model showing feature importance (mean absolute SHAP values) and the directional contribution of each metabolite to disease prediction across samples (beeswarm plot). The results indicate that model predictions are driven by a limited number of dominant metabolites. SHAP analysis was performed using R (ver. 4.4.2). X421, Citric acid; X6290, ΔTomeprazone M670H05; X32317, ΔFaradiol laurate.

Supplementary Figure S3

Logistic Regression Model for Diagnosing Pancreatic Cancer Using Three Metabolites in Pancreatic Juice (Three-Variable Model)

Variable	OR	p_value	VIF	Nagelkerke_R2	AUC	Sensitivity	Specificity
(Intercept)	0.5386	0.3515	NA	0.560	0.8701	0.6364	0.7857
X11488	0.1583	0.2019	1.1477				
X1515	0.342	0.1214	1.1989				
X421	0.3543	0.1626	1.0594				

X11488, Oleoylglycerol
X1515, FA(18:2)+2O
X421, Ciric acid

Summary of Three-Fold Cross-Validation

Fold	AUC	Sensitivity	Specificity
Mean	0.7815	0.7870	0.8111
SD	0.1123	0.1773	0.1884
RSD(%)	14.3639	22.5303	23.2246
Overall	0.7532	0.5758	0.8571

ROC curve 1

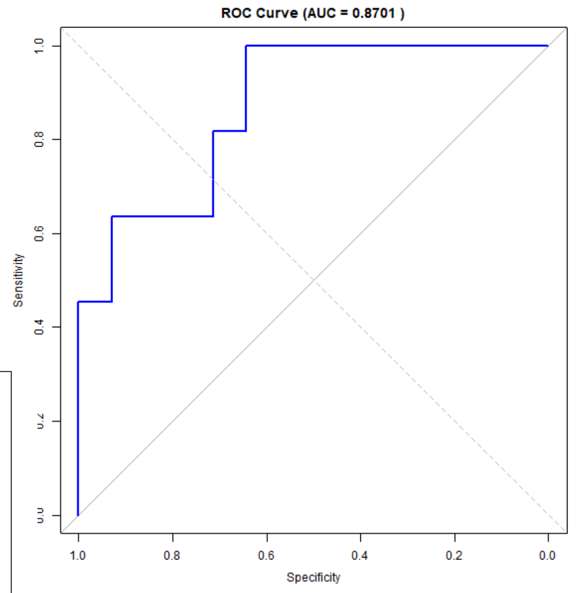

ROC curve 2

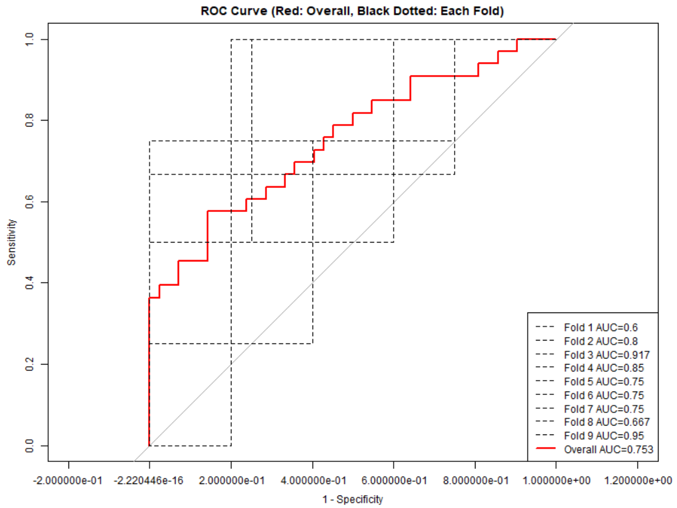

Supplementary Figure S3. Three-Variable Model for Pancreatic Cancer Diagnosis using Pancreatic Juice Metabolomics Data of Rank A-Annotated Metabolites

Model performance was optimized using LASSO logistic regression to select two variables from Rank A-annotated metabolites. The performance of the resulting two-variable model, along with its ROC curve (ROC Curve 1), based on data from all samples, is presented. Metabolomic data were obtained from pancreatic juice samples collected from patients. Model performance was validated through three-fold cross-validation within the discovery cohort. Nine ROC curves generated from three iterations of three-fold cross-validation are shown, along with the mean and variability of AUC, sensitivity, and specificity. These ROC curves are depicted as black dotted lines (ROC Curve 2), while the overall prediction ROC curve is shown as a solid red line. Because feature selection was performed outside the cross-validation framework—with CV applied only to the final model—the cross-validated performance is likely subject to optimistic bias due to data leakage. Model construction and validation were performed using R (version 4.4.2).

Supplementary Figure S4

X421
[M-H]-

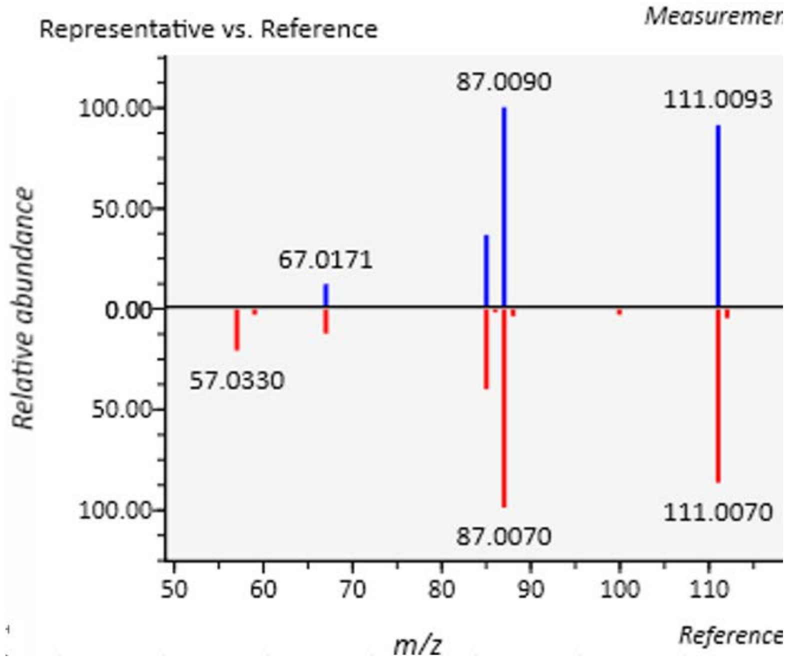

Citric acid

X6290
[M+H]+

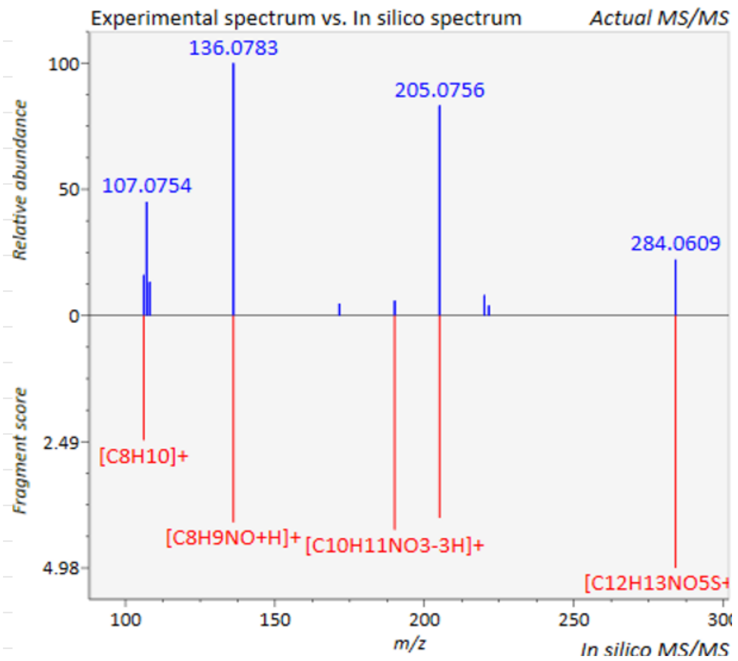

ΔTopramezone M670H05

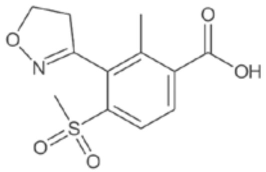

X32317
[M+H]+

No MS2 data
MS1= 625.5503

C42H72O3
ΔFaradiol laurate

Supplementary Figure S4. Compound Annotations for Pancreatic Juice Metabolites in the Logistic Regression Models

Compound annotations were performed based on MS/MS spectra using MS-DIAL (ver. 5.1), MS-FINDER (ver. 3.56), and publicly available metabolite databases such as HMDB. Metabolite X421 was annotated as citric acid due to a matched MS2 spectrum. Metabolite X4759 was annotated as Topramezone M670H05 based on its MS2 spectrum; however, since this compound is generally not a human metabolite, it was assigned Rank B. Metabolite X32317 was annotated as faradiol laurate (Rank B) based on its MS1 value.

Supplementary Figure S4

X11488
[M+H]+

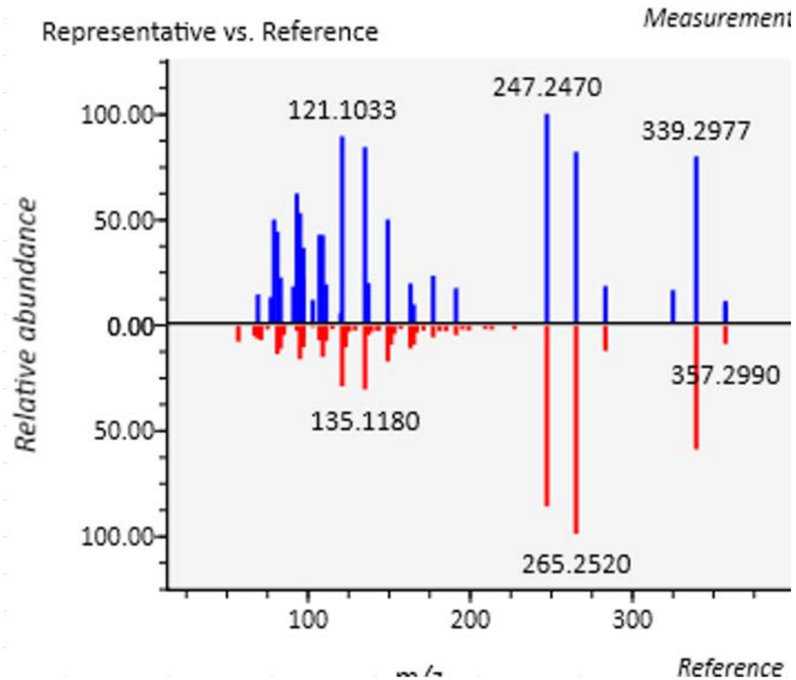

X1515
[M-H]-

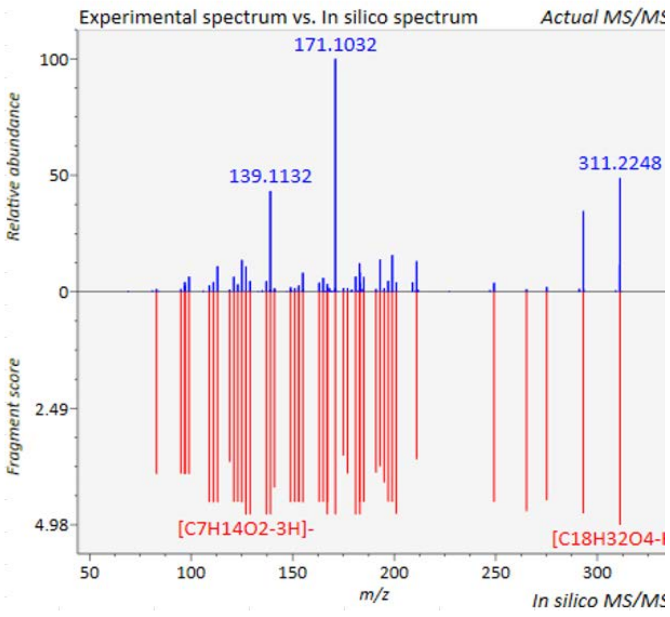

Continued from Supplementary Figure S4

Metabolite X11488 was annotated as oleoylglycerol based on a matched MS2 spectrum. Metabolite X1515 was annotated as FA(18:2)+2O, as its MS2 spectrum matched that of 9,10-DiHODE, a known component of FA(18:2)+2O.

Oleoylglycerol

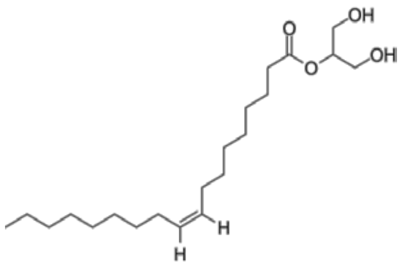

9,10-DiHODE
FA(18:2)+2O

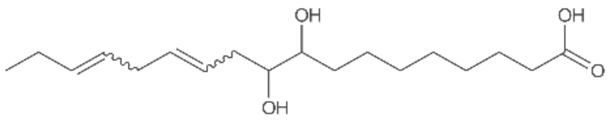

Supplementary Figure S5

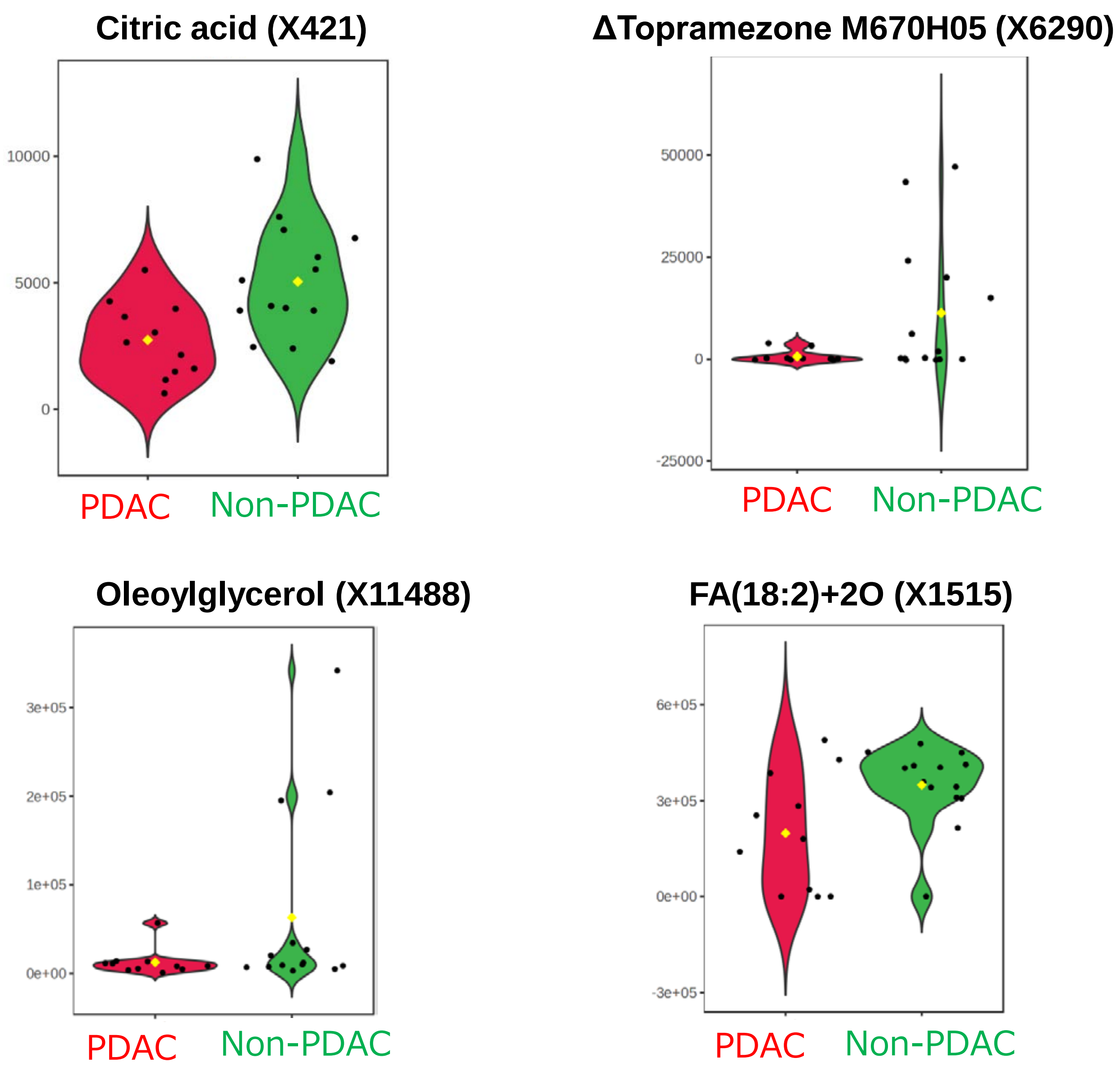

Supplementary Figure S5.
Violin Plots for Pancreatic Juice Metabolites in the Logistic Regression Models

Figures were generated using MetaboAnalyst (ver.6.0).
The vertical axis represents peak intensity.
PDAC: Group A
Non-PDAC: Group B
